# Evolution of assortative mating following selective introgression of pigmentation genes between two *Drosophila* species

**DOI:** 10.1002/ece3.8821

**Published:** 2022-04-13

**Authors:** Jean R. David, Erina A. Ferreira, Laure Jabaud, David Ogereau, Héloïse Bastide, Amir Yassin

**Affiliations:** ^1^ Laboratoire Évolution, Génomes, Comportement et Écologie CNRS IRD Université Paris‐Saclay – Institut Diversité Ecologie et Evolution du Vivant (IDEEV) Gif‐sur‐Yvette France

**Keywords:** experimental speciation, genome mapping, hybridization, mate choice, pigmentation

## Abstract

Adaptive introgression is ubiquitous in animals, but experimental support for its role in driving speciation remains scarce. In the absence of conscious selection, admixed laboratory strains of *Drosophila* asymmetrically and progressively lose alleles from one parental species and reproductive isolation against the predominant parent ceases after 10 generations. Here, we selectively introgressed during 1 year light pigmentation genes of *D*. *santomea* into the genome of its dark sibling *D*. *yakuba*, and vice versa. We found that the pace of phenotypic change differed between the species and the sexes and identified through genome sequencing common as well as distinct introgressed loci in each species. Mating assays showed that assortative mating between introgressed flies and both parental species persisted even after 4 years (~60 generations) from the end of the selection. Those results indicate that selective introgression of as low as 0.5% of the genome can beget morphologically distinct and reproductively isolated strains, two prerequisites for the delimitation of new species. Our findings hence represent a significant step toward understanding the genome‐wide dynamics of speciation‐through‐introgression.

## Graphical abstract

Adaptive introgression is widespread in nature, but its role in driving species remains contested. We showed that experimental introgression of 2–3 pigmentation loci between two *Drosophila* species with contrasting colors produced hybrid strains that were morphologically and behaviorally distinct from the parental species.

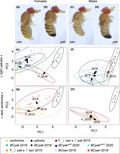

## 1 INTRODUCTION

In sexually reproducing organisms, speciation begins when extrinsic or intrinsic barriers significantly reduce gene flow between populations and ends with the evolution of pervasive phenotypic differences delimiting the nascent species (Coyne & Orr, 2004; Kulmuni et al., 2020; The Marie Curie SPECIATION Network, 2012). The pace of this process can be dramatically accelerated if the diagnostic characters also contribute, either directly or through genetic linkage, to reproductive isolation. The search for such traits, which were dubbed “magic,” has been a “holy grail” in speciation genetics (Martin & Richards, 2019; Servedio et al., 2011; Smadja & Butlin, 2011; Thibert‐Plante & Gavrilets, 2013). However, how such traits form is enigmatic, and theory predicts that substantial degrees of geographical isolation and long times of divergence are necessary for the build‐up of genetic barriers to reproduction (Richards et al., 2019). Therefore, it has been argued that adaptive introgression, that is, the exchange of beneficial alleles between species with intermediate levels of reproductive isolation (Hedrick, 2013), could significantly shorten the time of speciation. Introduced alleles could epistically interact with the host genome leading to the rapid formation of populations that are phenotypically distinct and reproductively isolated from the parental species (Abbott et al., 2013; Payseur & Rieseberg, 2016; Richards et al., 2019; Schumer et al., 2014). In spite of the growing evidence for the ubiquity of interspecific gene flow unraveled by recent comparative genomic studies in plants and animals (Edelman et al., 2019; Lamichhaney et al., 2015; Leducq et al., 2016; Pease et al., 2016; Racimo et al., 2015; Schumer et al., 2018), experimental tests for the role of adaptive introgression in the evolution of reproductive barriers are rare. Indeed, two recent reviews on experimental speciation had barely addressed the question of adaptive introgression (Fry, 2009; White et al., 2020).

For nearly 100 years, *Drosophila* species have been a primary model for the experimental study of speciation (Castillo & Barbash, 2017; Mallet, 2006). Introgression between species with incomplete reproductive isolation has long been used to identify the quantitative trait loci (QTL) responsible for phenotypic differences and reproductive barriers (e.g., Ding et al., 2016; Massey et al., 2021; Shahandeh & Turner, 2020; Tanaka et al., 2015). In those experiments, two species are crossed and their fertile F_1_ hybrid females are backcrossed to one parental species for one or a few generations. Introgressed genomic regions are then assessed using molecular markers and isogenic lines are produced via inbreeding to test for the statistical association with the phenotype of interest. Such short‐term introgression does not inform us much on how introgression can lead to the origin of new species. Indeed, whereas F_1_ hybrid males are sterile, a proportion of males issued from the first backcross are often fertile. When those males are left to mate with the backcross females, the proportion of sterile males progressively diminish each generation. In the absence of conscious selection on a particular introgressed phenotype, alleles from one parent, usually the one that was not used in the backcross, are gradually purged out in less than 20 generations (Amlou et al., 1997; David et al., 1976; Matute et al., 2020). Contrary to those experimental observations, comparative genomics studies have unraveled strong evidence for genetic introgression between many *Drosophila* species pairs (Lohse et al., 2015; Mai et al., 2020; Schrider et al., 2018; Turissini & Matute, 2017), with the traces of introgression sometimes persisting for millions of years (Suvorov et al., 2022).

To test for the effect of adaptive introgression on speciation, one should identify an easily measurable phenotype distinguishing a pair of species, deliberately select it in backcross flies for several generations, and then quantify the degree of reproductive isolation of introgressed flies with both parental species. Unfortunately, most sister *Drosophila* species are usually recognizable only on the basis of subtle differences in their genitalia whose dissection and measuring are quite difficult and laborious (Yassin, 2021). A striking exception is the case of the species pair of *D*. *yakuba* and *D*. *santomea*, which, in addition to genital differences, also shows a contrasting pigmentation pattern (Lachaise et al., 2000). Both species lack the characteristic sexual dimorphism of pigmentation found in all other species of the *melanogaster* subgroup, where the last abdominal segments of the females are lighter than those of the males. Those segments are equally dark or equally light in both sexes of *D*. *yakuba* and *D*. *santomea*, respectively. Both species can mate readily in the laboratory, producing fertile hybrid females but sterile males, and there is strong evidence from field studies and population genomics that hybridization takes place also in the wild on the island of Sao Tomé where *D*. *santomea* is endemic (Cariou et al., 2001; Llopart et al., 2005, 2014; Turissini & Matute, 2017). Leveraging the crossability of the two species, short‐term introgression experiments were used to identify the QTL underlying their morphological differences (Carbone et al., 2005; Coyne et al., 2004; Liu et al., 2019; Nagy et al., 2018; Peluffo et al., 2015) and reproductive isolation (Cande et al., 2012; Moehring et al., 2006a, 2006b). Introgressing dark pigmentation alleles of *D*. *yakuba* in the genome of the lightly pigmented *D*. *santomea* indicated that at least 5 loci were responsible for the striking pigmentation difference, namely the melanin‐synthesis genes *yellow* (*y*), *tan* (*t*) and *ebony* (*e*) and the transcription factors *Abdominal*‐*B* (*Abd*‐*B*) and *POU*‐*domain motif 3* (*pdm3*) (Liu et al., 2019). Remarkably, long‐term introgression experiments between *D*. *santomea* and *D*. *yakuba* showed, that in the absence of conscious selection on any of their morphological differences, reproductive isolation with the parental species may persist for 10 generations (Comeault & Matute, 2018), but at generation 20, introgressed flies completely resemble their *D*. *yakuba* parent with no trace of isolation (Matute et al., 2020).

In 2015, our late colleague Jean R. David (1931–2021) started two long‐term introgression experiments. In the first one, he deliberately introgressed light *D*. *santomea* alleles in the genome of dark *D*. *yakuba*, whereas in the second experiment he performed the opposite introgression, that is, introgressing dark *D*. *yakuba* alleles in the genome of light *D*. *santomea*. In this paper, we report the progress of his 5‐year experiments and the results of sequencing two lines from the first experiment. We show through behavioral assays that introgression of as low as 0.5% of the genome has been sufficient to produce flies that were morphologically and behaviorally distinct from both parental species, even after 60 generations from the end of selection. We discuss the relevance of our work to the role of adaptive introgression in speciation.

## 2 MATERIALS AND METHODS

### 2.1 Generation of introgression lines

**FIGURE 1:**
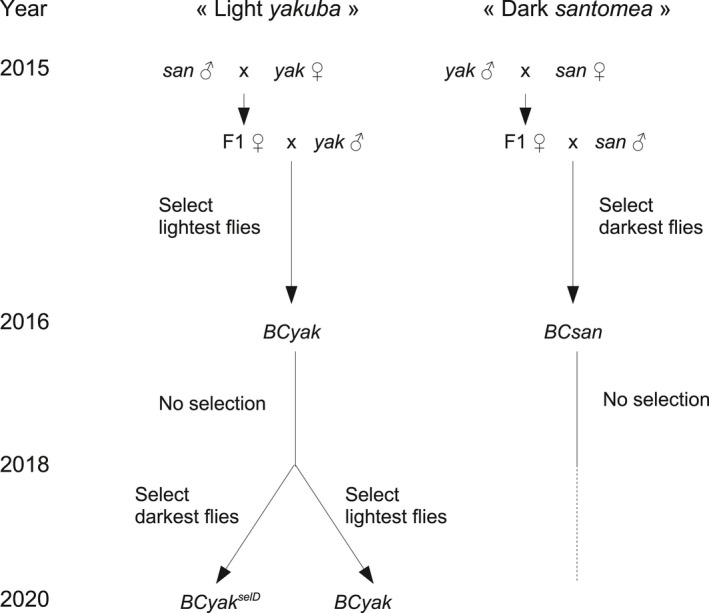
Timeline of the “light *yakuba*” and “dark *santomea*” introgression experiments showing the origin of the introgression strains for which pigmentation was scored

Two experiments were conducted from reciprocal crosses between a strain of *D*. *yakuba*, which was collected by L. Tsacas from Kounden, Cameroon in 1966, and *D*. *santomea* from the type laboratory strain collected by D. Lachaise from Sao Tomé Island in 1998. Strains and experimental lines were reared at 21°C on a standard *Drosophila* medium kept in culture bottles at a density of ~1,000 flies. The timeline of each introgression experiment is presented in Figure 1.

For the “light *yakuba*” experiment: virgin *D*. *yakuba* females were crossed to *D*. *santomea* males. Fertile F_1_ females were mated to *D*. *yakuba* Kounden males, and the progeny called backcross to *yakuba* (*BCyak*). Backcross flies contained a small proportion (not determined) of fertile males. Those flies were used as a mass population to produce a self‐reproducing strain. After a second generation of mass culture, phenotypes were observed on anesthetized, 3–5 days old flies, and we assumed that most females had already copulated, many of them with fertile males. Selection was made on females only, who were far more variable than males. At each generation ~50 females with the lightest phenotype were transferred to lay eggs in new culture bottles. Precise phenotypic measurements were not done on regular basis and the progress of selection (if any) was not monitored. However, from our empirical observations, the selection was not efficient; each generation, the light females produced the same proportion of light and dark flies. This result persisted for more than a year (~15 generations). Then, some positive effects were observed: pigmentation of the females became lighter, and also some effects were found on the males, who also could be selected, leading to the establishment of an introgressed *D*. *yakuba* strain in 2016 (hereafter *BCyak*), quite lighter than the typical *D*. *yakuba*, especially for the females. However, after 2 years from the end of selection, female dark pigmentation slightly increased, attaining the levels of those found in F_1_ hybrids. So a second round of selection on both males and females restarted in 2018, leading to two new derived introgression strains denoted *BCyak^CC^* and *BCyak^selD^* for flies selected for their light and dark abdomen, respectively.

For the “dark *santomea*” experiment: virgin *D*. *santomea* females were crossed to *D*. *yakuba* males. The fertile F_1_ females were backcrossed to *D*. *yakuba* males, and the progeny was reared as a mass culture. Selection started by keeping females with a slightly dark abdomen, but the progress was very slow and took more than a year. Interestingly, the dark pigmentation of the males increased more rapidly than that of the females, and after about half a year males were also included in selection. In 2016, an introgressed *D*. *santomea* strain, darker than the typical *D*. *santomea*, especially for males, was established and denoted *BCsan*.

Throughout the introgression experiments, no samples were archived frozen or in alcohol for genome sequencing and subsequent behavioral assays. Following the perturbations related to the COVID‐19 pandemic lockdowns in early 2020, and the deterioration of Jean David's health later that year, only two strains, denoted *BCyak* and *BCsan* were present at the time of genome sequencing in December 2020 and behavioral assays. Those two strains along with those of the parental species were used for genome sequencing and subsequent mapping of introgressed loci. Sequencing revealed both strains to be predominated by the *D*. *yakuba* genome, sharing two introgressed *D*. *santomea* loci at genes known to affect pigmentation (see Results below). Because selection on dark *D*. *yakuba* alleles in a *D*. *santomea* background would not have only fixed light *D*. *santomea* alleles, we therefore hypothesized that the two strains were derived from the same “light *yakuba*” experiment. This was reconfirmed by checking their male genitalia, which were both of the “*yakuba*” type, in contrast to previous microscopic preparations of *BCsan* strain up to April 2020. A contamination occurring after this date has likely replaced *BCsan* with one of the *BCyak* lines. Because the two strains, *BCyak* and *BCsan*, had two and three fixed *D*. *santomea* loci (see Results below), the two strains were then denoted *BCyak*‐*2* and *BCyak*‐*3*, respectively.

### 2.2 Pigmentation scoring and genitalia dissection

Abdominal pigmentation was scored on parental species, reciprocal F_1_ hybrids and the introgression lines following the scoring scheme of David et al., 1990), that is, the width of black area at the posterior part of each tergite was visually scored by establishing 11 phenotypic classes from 0 (no black pigment) up to 10 (tergite completely black). Abdominal tergites 2–7 as well as tergite 8 (the epigynium) were considered for females and tergites 2–6 as well as tergite 9 (the epandrium) were considered for males. For the introgression lines, scoring was made in 2016 at the end of selection and then once each 2 years (i.e., in 2018 and 2020). For each strain, ≥4 days old, 10 females and 10 males were used. Pigmentation scores are provided in Table S1. All statistical analyses were conducted using R (R Core Team, 2016).

We also aimed to quantify subtle differences in pigmentation intensity between the two strains that were sequenced in 2020, that is, *BCyak*‐*2* and *BCyak*‐*3*. For this, flies were killed in 70% ethanol and wings and legs removed using a pair of forceps. Each fly was then individually placed on its left side in 2 ml 70% ethanol solution in an excavated glass block and photographed under a binocular Leica stereoscope provided with a digital camera connected to a computer. Flies were photographed and grayscale intensity was measured using ImageJ (Abramoff et al., 2004) after manually defining the contour of each abdominal tergite.

The two parental species differ in their male genital traits, with the most easily traceable character being the loss of a pair of hypandrial (sternite 9) bristles in *D*. *santomea* (Nagy et al., 2018). At the end of selection in 2016, we dissected the male genitalia of the introgression strains and found that the presence or absence of the hypandrial bristles followed the direction of the backcross, that is, present in *BCyak* and absent in *BCsan*. Male genitalia were then routinely dissected on a regular basis to guarantee the distinction between the lines of the two experiments.

### 2.3 Genome sequencing and analysis of two introgressed BCyak strains

For the two strains *BCyak*‐*2* and *BCyak*‐*3*, genomic DNA was extracted from 30 flies using standard DNA extraction kit protocol Nucleobond AXG20 (Macherey Nagel 740544) with NucleoBond Buffer Set IV (Macherey Nagel 740604). DNA was then sequenced on Illumina Novaseq6000 platform (Novogene UK company limited). In order to update the current reference genome of *D*. *yakuba* v1.05 retrieved from Flybase (https://flybase.org/, Thurmond et al., 2019), we compared this version to a genome of the same *D*. *yakuba* strain that was sequenced and assembled using hybrid short‐read (Illumina) and long‐read (Oxford Nanopore) method (http://flyseq.org; Kim et al., 2021). We used assembly‐to‐assembly command in Minimap2 (Li, 2018) to generate a PAF file, based on which we attributed each new ≥100 kb‐long contig to the corresponding 1.05 chromosomal arm according to the longest homology tract. We also mapped each coding DNA sequence (CDS) to the new contigs using Blast (Altschul et al., 1997) in order to localize previously unmapped 1.05 contigs and genes. For each chromosome, assembled scaffolds were then ordered according to the cytological map of *D*. *yakuba* in (Lemeunier & Ashburner, 1976). This resulted into a newly assembled reference genome of *D*. *yakuba* (cf. Table S2) that we used for mapping introgressed loci.

Minimap2‐generated SAM files were converted to BAM format using samtools 1.9 software (Li et al., 2009). The BAM files were then cleaned and sorted using Picard v.2.0.1 (http://broadinstitute.github.io/picard/). We generated synchronized files for the 20 *D*.* y*.* yakuba* lines using Popoolation 2. We then used a customized Perl script to extrapolate allele frequencies to 2 diploid counts for each strain, after excluding sites with less than 10 reads and alleles with frequencies less than 25% for the total counts using a customized Perl script (cf. Ferreira et al., 2021). We also excluded tri‐allelic sites for each line. We then parsed the parental strains for divergent sites, that is, sites with distinct alleles fixed in each strain, and estimated the ancestry proportion at each site in the two introgressed strains in 50 kb‐long windows. All sequences were deposited in NCBI’s Sequence Read Archive (SRA) associated to the Bioproject (PRJNA820524).

### 2.4 Mating behavioral assays

We estimated precopulatory reproductive isolation between the two parental and the two introgressed strains, *Bcyak*‐*2* and *BCyak*‐*3*, using both no choice and two‐choice analyses for both sexes. For no choice analyses, 3–4 days old virgin males and females of all strains were introduced in pairs in individual food vials at around 9:00 AM and observed for 2 h. Mating pairs were counted for each mating pair. For each possible combination of pairs, 20 vials were tested. The proportion of successful matings in intraspecific pairs of *D*. *yakuba* was considered as the expected proportion, and a chi‐squared test comparing the observed proportions of successful mating involving an introgressed and a parental fly for each interstrain combination.

Two‐choice analyses were conducted for both males and females. For a given sex, a virgin fly was introduced into an individual vial along with two virgin flies from the opposite sex, with one being from the same strain as the tested fly and one from another strain. Copulations were observed also for 2 h, and once copulation started flies were anesthetized under slight CO_2_, and the identity of the mating and the un‐mating flies identified. In some instances, for example, those involving a *D*. *santomea* male, no marking was needed. For most other cases, flies were individually left to feed in vials with artificial food blue or red colorants (Sainte Lucie co., France) 24 h before the start of the experiment as in Comeault and Matute (2018). A chi‐squared test was then conducted for each strain pairing to test the deviation from parity between homo‐ and hetero‐gamic successful matings.

For all behavioral analyses, flies were maintained in a temperature‐regulated fly room with glass windows, that is, with natural cycles of night and day. Copulations were conducted on lab benches under light conditions. Previous experimentations (Llopart et al., 2002) showed no differences in mating choice between *D*. *yakuba* and *D*. *santomea* under light and dark conditions.

## 3 RESULTS

### 3.1 Experimental hybridization led to sexually dimorphic, phenotypically distinct introgression lines

**FIGURE 2:**
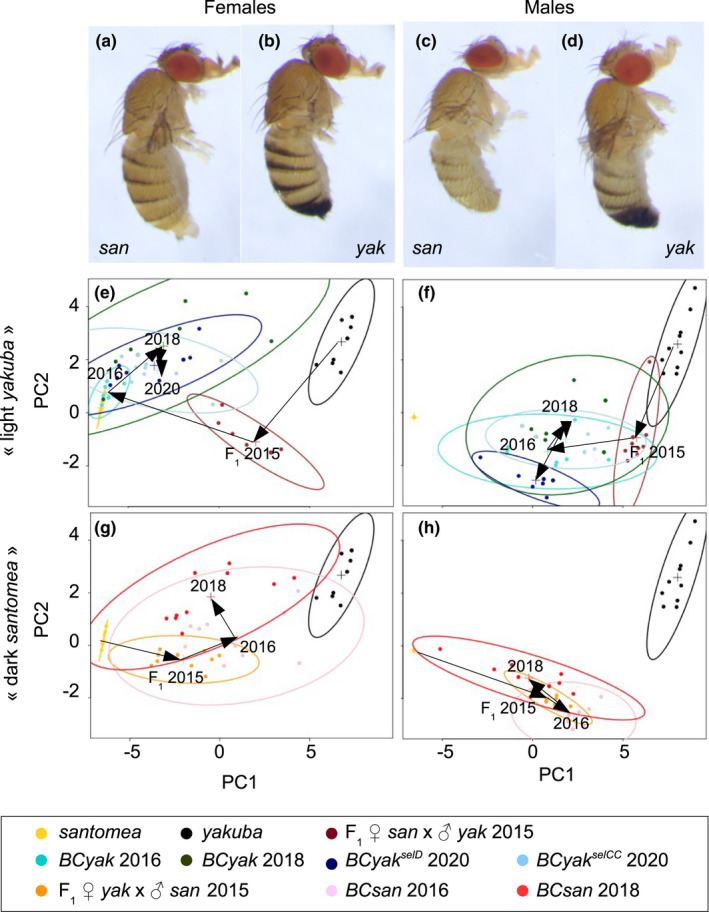
(a–d) Photomicrographs of females and males of the parental species, light *Drosophila santomea* (a, c) and dark *D*. *yakuba* (b, d). (e–h) Pigmentation introgression trajectories in the “light *yakuba*” (e, f) and the “dark *santomea*” (g, h) experiments. (e–h) Principal component analysis (PCA) of pigmentation scores on six successive abdominal segments per individual was conducted on combined males and females data but each sex per experiment was presented in a separate panel according to the coordinates of the two first principal components. In each panel, 95% confidence ellipses for the two parental species are shown in yellow (*D*. *sanromea*) and black (*D*. *yakuba*). Colors refer to F_1_ hybrids issued from the cross between female *yakuba* × male *santomea* (brown), *BCyak^2016^* (turquoise), *BCyak^2018^* (dark green), *BCyak^selD_2020^* (dark blue), *BCyak^CC_2020^* (light blue), F_1_ hybrids issued from the cross between female *santomea* x male *yakuba* (orange), *BCsan^2016^* (pink) and *BCsan^2018^* (red). Arrows indicate the trajectory of pigmentation changes in each panel

The trajectories of pigmentation evolution during the two 5‐year introgression experiments are given in Figure 2 in terms of the PCA of pigmentation scores. The first principal component (PC1) explained 75% of the variance. It mostly correlated with the pre‐penultimate and penultimate segments (i.e., segments 6 and 7 in females and 5 and 6 in males) with *r* = .56 and .78, respectively. The second principal component (PC2) explained 13% of the variance, and it mostly correlated with the ultimate segment of the body (i.e., the female epigynium and the male epandrium) with *r* = .81. The trajectories differed according to the direction of selection and the sex.

At the end of selection in 2016, introgressed “light *yakuba*” females (Figure 2e) were much lighter than the parental *D*. *yakuba* (*t* test for the sum of segments 6 and 7 = 56.65, *p* < 2.2 × 10^−16^). They almost resembled *D*. *santomea* females, although they were still darker from the later species (*t* = 2.59, *p* = .029). Interestingly, all the segments were quite similar, and the last one, that is, the epigynium or tergite 8, which is very dark in *D*. *yakuba* was the lightest in the introgressed females (*t* = 23.24, *p* < 2.4 × 10^−9^). The posterior segments of introgressed males (Figure 2f) were lighter than *D*. *yakuba* (*t* test for the sum of segments 5 and 6 = 9.25, *p* < 4.3 × 10^−6^) but still much darker than *D*. *santomea* (*t* = 10.85, *p* < 1.8 × 10^−6^). However, the last segment, that is, the epandrium or tergite 9, became almost completely light (*t* = 10.16, *p* < 1.7 × 10^−6^), as in *D*. *santomea* (*t* = 1.00, *p* = .34). For the “dark *D*. *santomea*” experiment, introgressed females (Figure 2g) at the end of selection in 2016 were darker than the parental *D*. *santomea* (*t* test for the sum of segments 6 and 7 = 10.11, *p* < 3.3 × 10^−6^), but not as dark as *D*. *yakuba* (*t* = 7.60, *p* < 1.8 × 10^−5^). The males (Figure 2h), on the other hand, had much darker posterior abdomen (*t* test for the sum of segments 5 and 6 = 21.34, *p* < 5.1 × 10^−9^), yet still lighter than *D*. *yakuba* (*t* = 10.96, *p* < 4.1 × 10^−7^). The last segments in both sexes were completely light as in *D*. *santomea*. Remarkably, introgressed females from both experiments significantly differed (*t* = 9.46, *p* < 3.5 × 10^−6^), but not introgressed males (*t* = 1.99, *p* = .065).

After 2 years from the end of selection in 2016, both experiments tended toward pigmentation values of the ancestral backcross parent, but at a much slower rate. This was most pronounced in females of the “light *yakuba*” experiment (*t* = 2.79, *p* = .021), but not in males (*t* = 1.02, *p* = .321), and in males of the “dark *santomea*” experiment (*t* = 3.42, *p* < .004), but not in females (*t* = 1.63, *p* = 0.121). For the second round of selection in the “light *yakuba*” experiment, starting in 2018, the two strains *BCyak^CC^* and *BCyak^selD^* very slightly differed only for male pigmentation of segments 5 and 6 in 2020 (*t* = 2.19, *p* = .042). This indicated that selection has attained its limits very rapidly in 2016, but morphological differences between introgressed flies and their parental species persisted for more than 60 generations after selection.

### 3.2 Two and three *D. santomea* loci were fixed in the two light *D. yakuba* strains

**FIGURE 3:**
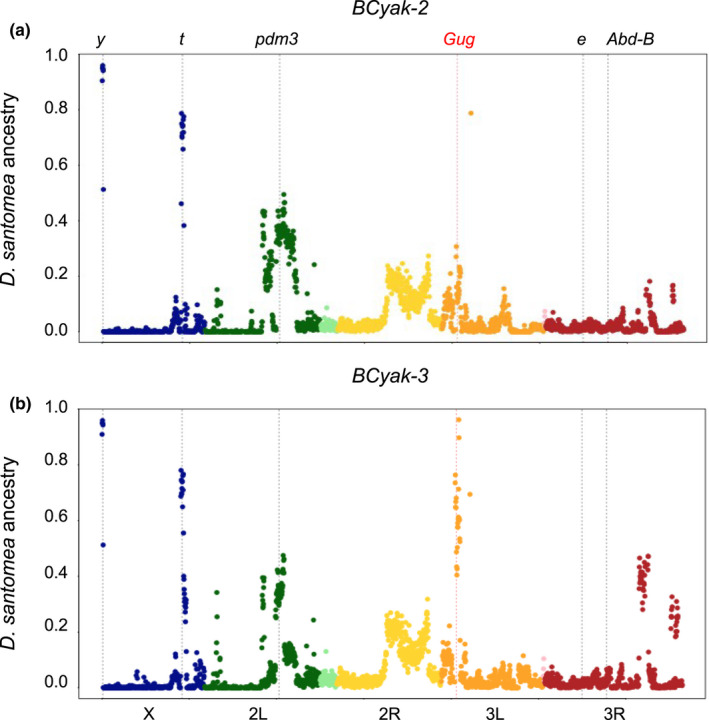
Proportion of *D*. *santomea* ancestry averaged over 50‐kb windows in two introgressed “light *yakuba*” lines (a) *BCyak*‐*2* and (b) *BCyak*‐*3*. Vertical dotted lines refer to the location of the five pigmentation genes that were identified in Liu et al.'s (2019) “dark *santomea*” investigation (in black) as well as the location of the transcription factor *Gug* (in red)

As stated in the Materials and Methods, we sequenced in December 2020 the genome of the two remaining introgressed strains in the laboratory, which were named *BCyak* and *BCsan*. We then estimated the ancestry proportion of both parental species across the genome. This showed that both strains belonged to the “light *yakuba*” experiments, bearing only 5%–6% alleles from *D*. *santomea*. The two strains showed almost the same profile of *D*. *santomea* introgression tracts, which were classified either as fixed or nearly fixed (*D*. *santomea* ancestry ≥75%) and intermediate (*D*. *santomea* ancestry ≥40%) (Table 1; Figure 3). The two strains were called *BCyak*‐*2* and *BCyak*‐*3* in reference to the number of fixed or nearly fixed introgression loci.

**TABLE 1:** Coordinates according to the *Drosophila yakuba* reference genome v.1.05 of *D*. *santomea* loci that were fixed (F) or segregate at intermediate frequencies (I) in introgressed light *D*. *yakuba* strains

Locus	Length	*BCyak‐2*	*BCyak‐3*	No. of genes	Candidate(s)
X:15,000–226,000	211 kb	F	F	22	*y*
X:17,395,000–17,967,000	572 kb	F	F	49	*t*
2L:16,511,000–18,064,000	1,553 kb	I	I	253	*pdm3*
3L:3,160,000–4,086,000	926 kb	‐‐‐	F	168	*Gug*
3R:19,079,000–21,169,000	2,090 kb	‐‐‐	I	304	

For *BCyak*‐*2* (Figure 3a), the two fixed loci were both X‐linked, each centering on one major melanin‐synthesis gene, namely *y* and *t*. A third peak with intermediate frequencies was also present on chromosomal arm 2L and it centered on the *pdm3* transcription factor gene. All of those genes, *y*, *t*, and *pdm3*, were found in the opposite experiment by Liu et al. (2019) who introgressed dark *D*. *yakuba* alleles into *D*. *santomea*.

The *BCyak*‐*3* strain had exactly the same introgression profile as *BCyak*‐*2*, that is, fixed *y* and *t* loci and intermediate *pdm3* locus (Figure 3b). However, it had also two differences. First, a locus on chromosomal arm 3L had a high proportion of *santomea* alleles and nearly reached fixation. A second locus on chromosomal arm 3R also had high, yet intermediate proportions. None of those two loci harbors any of the previously identified genes known to affect pigmentation differences between *D*. *santomea* and *D*. *yakuba* (Liu et al., 2019). However, the 3L locus centered on a transcription factor, *Grunge (Gug)*, which controls the expression of *t* and *e* in *D*. *melanogaster* (Rogers et al., 2014), and it is therefore a candidate pigmentation locus. There are no candidate pigmentation genes in the 3R locus with intermediate frequency in *BCyak*‐*3*.

**FIGURE 4:**
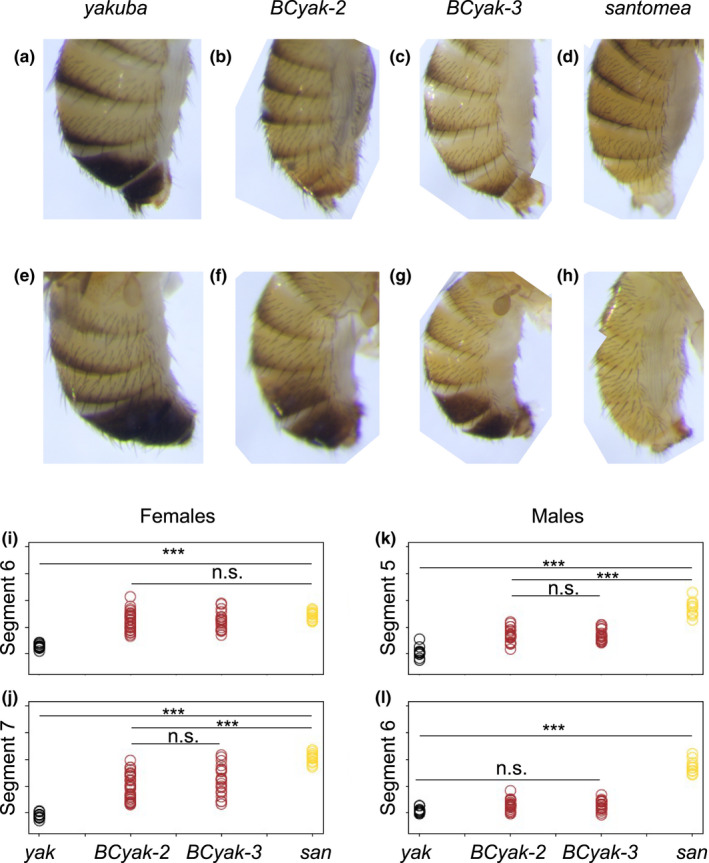
(a–h) Photomicrographs of abdominal pigmentation in males and females of the parental species, *D*. *yakuba* and *D*. *santomea*, and the two introgressed “light *yakuba*” lines, *BCyak*‐*2* and *BCyak*‐*3*. (i–l) grayscale intensity of females’ abdominal segments 6 and 7 and males’ abdominal segments 5 and 6. Tukey's HSD significance level: *<.05, **<.01 and ***<.001

The two strains were likely derived from the *BCyak^CC^* and *BCyak^selD^* strains, which corresponded to the second round of selection in the “light *yakuba*” experiment, and which by 2020 slightly differed in male pigmentation (see above). However, the two sequenced strains, *BCyak*‐*2* and *BCyak*‐*3*, did not show significant difference in pigmentation, even when more numerical analyses were used to quantify melanization (Figure 4). Nonetheless, both strains showed significant differences with the two parental species for females’ segment 7 and males’ segment 5, and from a single parent for females’ segment 6 and males’ segment 6, resembling *D*. *santomea* for the former and *D*. *yakuba* for the later.

### 3.3 Assortative mating between introgressed strains and parental species

In no‐choice experiments, homogamic mating occurred with almost the same frequency between pairs belonging to the same strain/species (70–85%) (Table 2). The two introgressed *yakuba* lines, *BCyak*‐*2* and *BCyak*‐*3*, readily mated with each other. However, a significant low mating success was observed in the cross between *D*. *yakuba* females and *BCyak*‐*3* males. Interspecific crosses between *D*. *santomea* and *D*. *yakuba*, as well as between *D*. *santomea* females and males from both introgressed lines were significantly low. Remarkably, more successful heterogamic matings were observed in cases involving *D*. *santomea* males and females from the introgressed *yakuba* lines who have lighter abdomen compared to *D*. *yakuba*.

**TABLE 2:** No choice experiment within and between pure parental species, *Drosophila yakuba* and *D*. *santomea*, and two introgressed “light *yakuba*” strains

Males	Females			
	*yakuba*	*BCyak‐2*	*BCyak‐3*	*santomea*
*yakuba*	17	17	14	2 (***)
*BCyak‐2*	15	14	12	1 (***)
*BCyak‐3*	12 (**)	15	16	0 (***)
*santomea*	2 (***)	8 (***)	8 (***)	15

Note

Twenty copulating pairs were tested per cross. For heterogamic crosses, significant deviation from the homogamic *D*. *yakuba* cross, that is, 17 successful crosses out of 20, was estimated using chi‐squared test: *<.05, **<.01, and ***<.001.

For choice experiments, all crosses involving *D*. *yakuba* and the introgressed lines on the one hand and *D*. *santomea* on the other hand were significantly homogamic, regardless of the tested sex (Table 3). However, sex‐dependent assortative mating was found for all crosses between *D*. *yakuba* and introgressed strains. In all those crosses, females always showed a higher preference for homogamic males, whereas no significant departure from parity was observed for males.

**TABLE 3:** Two‐choice mating preference experiments

Cross	Female choice				Male choice			
	*N*	Strain1	Strain2	F.E.T.	*N*	Strain1	Strain2	F.E.T.
		*yakuba*	*BCyak‐2*			*yakuba*	*BCyak‐2*	
*yakuba*	30	12	6		30	17	9	
*BCyak‐2*	40	7	16	*	30	11	10	n.s.
		*yakuba*	*BCyak‐3*			*yakuba*	*BCyak‐3*	
*yakuba*	40	22	7		30	9	7	
*BCyak‐3*	35	11	15	*	53	19	11	n.s.
		*yakuba*	*santomea*			*yakuba*	*santomea*	
*yakuba*	28	12	0		25	13	3	
*santomea*	30	0	23	***	30	0	22	***
		*BCyak‐2*	*BCyak‐3*			*BCyak‐2*	*BCyak‐3*	
*BCyak‐2*	30	12	4		50	17	11	
*BCyak‐3*	30	5	12	*	30	10	14	n.s.
		*BCyak‐2*	*santomea*			*BCyak‐2*	*santomea*	
*BCyak‐2*	20	13	0		30	12	7	
*santomea*	20	2	16	***	37	2	21	***
		*BCyak‐3*	*santomea*			*BCyak‐3*	*santomea*	
*BCyak‐3*	50	22	4		30	13	6	
*santomea*	20	0	16	***	30	2	21	***

Note

F.E.T. = significance level of Fisher's exact test for homogamy in each possible combination: *<.05, **<.01 and ***<.001.

## 4 DISCUSSION

We reported here the results of 5‐year experiments to reciprocally introgress genes causing morphological difference between a pair of sister species with a major difference in body pigmentation, and a strong, yet incomplete reproductive isolation. We showed that such introgression was possible and that the limits of selection were attained within only a single year (~15 generations), with the new phenotypes of the introgressed flies remaining distinct from the parental species. Remarkably and contrary to previous studies with no conscious selection on a morphological trait (Amlou et al., 1997; David et al., 1976; Matute et al., 2020), assortative mating persisted in the introgressed flies even after 4 years from the end of selection (~60 generations). The success of selective introgression might strongly depend on the nature of the phenotype. Pigmentation can easily be scored and measured and its variation often has a simple, oligogenic architecture (Massey & Wittkopp, 2016). By contrast, when Amlou et al. (1997) tried to introgress resistance to a fruit toxin from *D*. *sechellia* into *D*. *simulans*, their attempt failed, likely due to the difficulty of measuring toxicity and to the polygenic nature of survival as a phenotype. Indeed, many known cases of cross‐species adaptive introgression involve color variation, for example, coat in wolves (Anderson et al., 2009), skin and hair colors in humans (Dannemann & Kelso, 2017), wing patterns in mimetic butterflies (Edelman et al., 2019), winter‐coats in hares (Giska et al., 2019), plumage in pigeons (Vickrey et al., 2018) and wagtails (Semenov et al., 2021), and beaks in Darwin's finches (Enbody et al., 2021).

A parallel dynamics of introgressed trait trajectories was observed in both experiments, characterized by an initial phase of slow progress of introgression during selection. This progress was most likely due to the nature of the trait, that is, pigmentation is a complex trait with major epistatic and dominance interactions, and the efficiency of selection being applied to a single sex, the female. Male sterility tends to decrease across successive generations, as introgressed incompatibility genes are selected against. Because selection was conducted on females that were presumably mated, it is likely that fertile males bearing the ancestral phenotype have sired the progeny of those females. In agreement with this hypothesis, and with our knowledge of the major contribution of the X chromosome to pigmentation differences between the parental species (Carbone et al., 2005; Liu et al., 2019; Llopart, Elwyn, Lachaise, et al., 2002; Figure 3), selected light *yakuba* females continued to sire equal proportions of light and dark et al flies (see Materials and Methods). Positive results occurred most likely when successive recombination started to dissociate pigmentation and incompatibility loci, although we still lack the knowledge of the strength of their linkage.

Introgressed flies differed from their parents in both the degree of pigmentation but also in resuscitating ancestral sexual dimorphism that was independently lost in the parental species. Because of the major effect of the X chromosome on pigmentation differences between *D*. *yakuba* and *D*. *santomea*, F_1_ hybrid females had intermediate phenotypes whereas males resembled those from the maternal species. This sexual dimorphism in the hybrids persisted throughout the selection experiments and even after the end of selection. If the loss of sexual dimorphism in *D*. *santomea* and *D*. *yakuba* has involved different sex‐specific regulatory changes affecting similar sets of melanin‐synthesis genes, introgression of those changes in the new backgrounds could epistatically resuscitate the lost dimorphism. We were not able to sequence our introgressed “dark *santomea*” flies which were lost by mid‐2020, but fortunately Liu et al. (2019) have conducted similar experiment and identified at least five genes whose *D*. *yakuba* alleles darken *D*. *santomea* male pigmentation. Our introgressed loci in the “light *yakuba*” flies overlapped with three out of these genes, namely the X‐linked melanin‐synthesis genes *y* and *t* and the autosomal transcription factor *pdm3*. By contrast, we did not detect signal of introgression on either the melanin‐synthesis gene *e* or the homeotic transcription factor *Abd*‐*B*, which were identified in “dark *santomea*” (Liu et al., 2019). This was in agreement with Liu et al.’s (2019) observations. *Abd*‐*B*, which has lower expression in *D*. *santomea*, does not affect *D*. *santomea* pigmentation genes due to *cis*‐regulatory mutations of its melanin‐synthesis genes. Similarly, whereas *D*. *santomea e* has a higher expression associated with the insertion of a helitron in its regulatory sequence, the presence of the same *D*. *santomea* haplotype in *D*. *yakuba* does not affect its pigmentation (Liu et al., 2019).

The most intriguing result was the autosomal locus that was fixed or nearly fixed in only one of the two introgressed *BCyak* strains, and which was not identified by Liu et al. (2019) in their “dark *santomea*” flies. This locus contained the transcription factor *Gug*, which may have the opposite effect of *pdm3* on pigmentation intensity and sexual dimorphism. RNA interference (RNAi) silencing of this gene in the abdomen of *D*. *melanogaster* reduces pigmentation, with the reduction being more pronounced in males, whereas RNAi of *pdm3* increases pigmentation, with the increase being more pronounced in females (Rogers et al., 2014). Whereas *pdm3* is a suppressor of *y* in *D*. *santomea* (Liu et al., 2019), *Gug* is an enhancer of *t* and a suppressor of *e* in *D*. *melanogaster* (Rogers et al., 2014). Therefore, it is possible that the gain of female‐specific pigmentation in *D*. *yakuba* was partly due to a down‐regulation of *pdm3* whereas the loss of male‐specific pigmentation in *D*. *santomea* was partly due to a up‐regulation of *Gug*. The lack of significant difference in pigmentation between *BCyak*‐*2* and *BCyak*‐*3* argues against any role of the 3L locus, including *Gug*, on pigmentation. However, we note that pigmentation analysis of those two strains has been made in December 2021 after at least 18 months from the end of the second round of selection in the “light *yakuba*” experiment. Laboratory experiments and population analyses in *Drosophila* have suggested that balancing selection may act on pigmentation genes, hence restoring their alleles to intermediate frequencies when selection ends (Kalmus, 1945; L’Héritier & Teissier, 1937; Rendel, 1951). For example, pigmentation polymorphism in *D*. *kikkawai*, which is controlled by the *pdm3* locus (Yassin, Delaney, et al., 2016), is maintained by heterozygous advantage in experimental populations (Freire‐Maia, 1964). Similarly, ancient balancing selection on *t* was demonstrated in *D*. *erecta* (Yassin, Bastide, et al., 2016). Further isolation from *pdm3* and *t* of the introgressed locus on 3L and subsequent molecular dissection are therefore needed to understand its potential role in pigmentation evolution.

Color‐based assortative mating could lead to the loss of sexual dimorphism and ultimately precopulatory reproductive isolation. Our results showed that fixation of as low as 0.8 Mb (~0.5% of the genome) during selection on pigmentation loci has altered mating propensities between pure and introgressed flies. The demonstration of color‐based (dis)assortative mating in *Drosophila* has long been problematic (Kopp et al., 2000; Llopart et al., 2002). Our behavioral assays support the presence of color‐based assortative mating between *D*. *yakuba* and *D*. *santomea*, but in a way that was asymmetric between the sexes and dependent on the degree of divergence. On the one hand, light male *D*. *santomea* had almost 5‐fold success in mating with introgressed light *D*. *yakuba* females than with dark pure *D*. *yakuba* in no choice experiments. On the other hand, light females from both introgressed *BCyak*‐*2* and *BCyak*‐*3* showed preference for their own light males over pure dark *D*. *yakuba* males. This suggests that the two X‐linked *y* and *t* loci that were fixed in both strains probably play a role in color‐based assortative mating. However, female‐limited assortative mating also existed between the introgressed strains *BCyak*‐*2* and *BCyak*‐*3*, in spite of their great coloration resemblance. The fixed autosomal locus in *BCyak*‐*3* may therefore also contain elements affecting behavior. In addition to its possible effect on pigmentation, the transcription factor *Gug* also interacts with another transcription factor, *hairy (h*), which is also located in the same fixed locus, in affecting the size of male genital organs that are used to grasp the females during mating, namely the surstyli (claspers) (Hagen et al., 2021). The effect of pigmentation genes on mating behavior can be attained either directly through pleiotropy or indirectly genetic linkage to other mating phenotypes (Wellenreuther et al., 2014). Pleiotropy should drive more pervasive associations between pigmentation and mating behavior than linkage. A possible source of genetic linkage could have been the physical proximity in the low recombining subtelomeric region of the X chromosome between *y* and the enhancer of *scute (sc)* which led to the loss of the hypandrial bristles and gain of extranumerary sex comb teeth in *D*. *santomea* males (Nagy et al., 2018). Both characters may be involved in copulation and consequently contribute to mating success or choice. However, we found through regular dissections of the genitalia that this strong linkage was broken during the first year of the selection experiment, dissociating pigmentation, and hyprandial bristles.

In conclusion, our result demonstrate that selective introgression on a morphological phenotype could rapidly lead to the evolution of pervasive behavioral isolation. They hence complement previous *Drosophila* experimental speciation studies, which showed that adaptation from standing variation to contrasting environments could lead the evolution of reproductive isolation (Fry, 2009). Pigmentation also responds to diverse natural selection pressures (Bastide et al., 2014) including those that discriminate the ecological niches of *D*. *santomea* and *D*. *yakuba* such as temperature, desiccation, and UV intensity (Comeault & Matute, 2021; Matute & Harris, 2013; Matute et al., 2009). Further experimental manipulations, for example, testing competition between pure and introgressed flies in different environments, coupled with the investigation of postcopulatory isolation barriers, will definitively shed more light on genome dynamics of homoploid speciation in animals, hence bridging experimental studies with empirical field observations in a primary model.

## CONFLICT OF INTEREST

We declare no conflicts of interest.

## AUTHOR CONTRIBUTIONS

**Jean R. David:** Conceptualization (lead); Data curation (equal); Formal analysis (equal); Investigation (equal); Methodology (equal). **Erina A. Ferreira:** Data curation (equal); Formal analysis (equal). **Laure Jabaud:** Data curation (equal); Formal analysis (equal). **David Ogereau:** Data curation (equal); Formal analysis (equal). **Héloïse Bastide:** Data curation (equal); Formal analysis (equal); Supervision (equal). **Amir Yassin:** Conceptualization (supporting); Data curation (equal); Formal analysis (lead); Investigation; Methodology; Resources (equal); Supervision (equal); Validation (lead); Visualization (lead); Writing – original draft (lead); Writing – review & editing (lead).

## Supporting information

Table S1

Table S2

## Data Availability

All sequences were deposited in NCBI’s Sequence Read Archive (SRA) associated to the Bioproject (PRJNA820524).
