#  

**DOI:** 10.1002/ece3.8917

**Published:** 2022-05-03

In the recent article by David et al. (2022), the word “up‐regulation of Gug” should be replaced by "down‐regulation of Gug" in the fourth paragraph of Discussion section.

The authors apologize for the error.
